# Speckle tracking echocardiographically-based analysis of ventricular strain in children: an intervendor comparison

**DOI:** 10.1186/s12947-020-00199-x

**Published:** 2020-05-21

**Authors:** Alessandra M. Ferraro, Adi Adar, Sunil J. Ghelani, Lynn A. Sleeper, Philip T. Levy, Rahul H. Rathod, Gerald R. Marx, David M. Harrild

**Affiliations:** 1 Department of Cardiology, Boston Children’s Hospital, Boston, MA USA; 2 Department of Pediatrics, Harvard Medical School, Boston, MA USA

**Keywords:** Speckle tracking echocardiography, Strain, Time to peak standard deviation, Pediatric

## Abstract

**Background:**

Strain and synchrony can be calculated from a variety of software packages, but there is a paucity of data with inter-vendor comparisons in children. To test the hypothesis that different packages may affect results, independent of acquisition, we compared values obtained using two commercially available analysis tool (QLAB and TomTec), with several different settings.

**Methods:**

The study population included 108 children; patients were divided into three groups: (1) normal cardiac structure and conduction; (2) ventricular paced rhythm; and (3) flattened ventricular septum (reflecting right ventricular pressure or volume load lesions). We analyzed the same image acquired from the apical 4-chamber (AP4) and short-axis at the mid-papillary level (SAXM) views in both QLAB (versions 10.5 and 10.8) and TomTec (version 1.2). In QLAB version 10.8, low, medium, and high quantification smoothness settings were employed. In TomTec, images were analyzed with both low and high frame rates. Tracking quality for each package was graded. AP4 and SAXM strain and synchrony values were recorded. A mixed-effects linear regression model was used, with main effect considered significant if the *p*-value was < 0.05.

**Results:**

Tracking scores were high for all packages except QLAB 10.5 in the SAXM view. AP4 and SAXM strain values varied significantly between QLAB 10.5 and the other packages. Synchrony values varied widely for all strain values (*p* < 0.001 for both) in all packages. Quantification smoothness changes in QLAB 10.8 did not impact strain significantly in any patient group; temporal resolution changes in TomTec resulted in strain differences in children with flat ventricular septums, but not those with normal or ventricular paced hearts.

**Conclusion:**

Synchrony values varied substantially among all packages in children. Strain values varied widely between QLAB 10.5 and all other software packages, recommending avoidance of QLAB 10.5 for future studies. Quantification smoothness settings in QLAB 10.8 resulted in minimal strain differences. In TomTec, low and high frame rate strain values differed only in a subset of patients (flattened septum). These data suggest that reliable comparisons between strain values derived from QLAB and TomTec is possible in certain cases, but that caution should be used especially in different hemodynamics conditions.

## Introduction

Left ventricular (LV) strain by two-dimensional (2D) speckle-tracking echocardiography has been shown to be a reliable and clinically important method of quantitatively characterizing cardiac function in children [1–3]. In addition, LV strain is correlated with synchrony [3–6] and their interdependence plays an important role in managing patients with both congenital and acquired heart disease [7–12]. Strain and synchrony can be calculated with the use of a variety of analysis packages, some of which are vendor-specific, while others are vendor-independent [13]. Recent studies in adults [14] and children [15, 16], as well as an international task force for deformation imaging, have investigated the variations in reported values of strain from different packages [10, 13]. A paucity of evidence on inter-vendor synchrony differences currently exists, with only a single study published in adults, and none in children [4]. We hypothesized that different modern analysis packages may produce different results in both strain and synchrony values in children, independent of acquisition. Accordingly, the aim of this study was to evaluate the variability of analyses derived from speckle tracking echocardiography using three popular software platforms (QLAB 10.5, 10.8, and TomTec 1.2) for the measurement of longitudinal strain, circumferential strain, and synchrony.

## Methods

### Patient population and study design

We performed a single-center, retrospective analysis of data from pediatric patients (≤18 years old). The patients were chosen from three groups: (1) normal heart structure and function; (2) ventricular paced rhythm with a wide QRS (duration of ≥120 msec); (3) abnormal septal contour with flattened ventricular septum (in systole or diastole, due to either right ventricular pressure or volume-overload lesions). These groups were chosen to represent a broad variety of patterns of ventricular conduction. All patients had biventricular circulation and high-quality 2D echocardiographic images. Group 1 patients had been referred for echocardiography with an indication of murmur/ palpitations/ family history of congenital heart disease, and had been found to have normal heart structure, function, blood pressure for their age, and no evidence of pulmonary hypertension [3]. To ensure age diversity, equal numbers of patients were randomly selected from three age range cohorts for each clinical group: 0-6y; > 6-12y; > 12-18y. This study was approved by the Institutional Review Board at our institution.

### Echocardiographic image acquisition and analysis

**Fig. 1:**
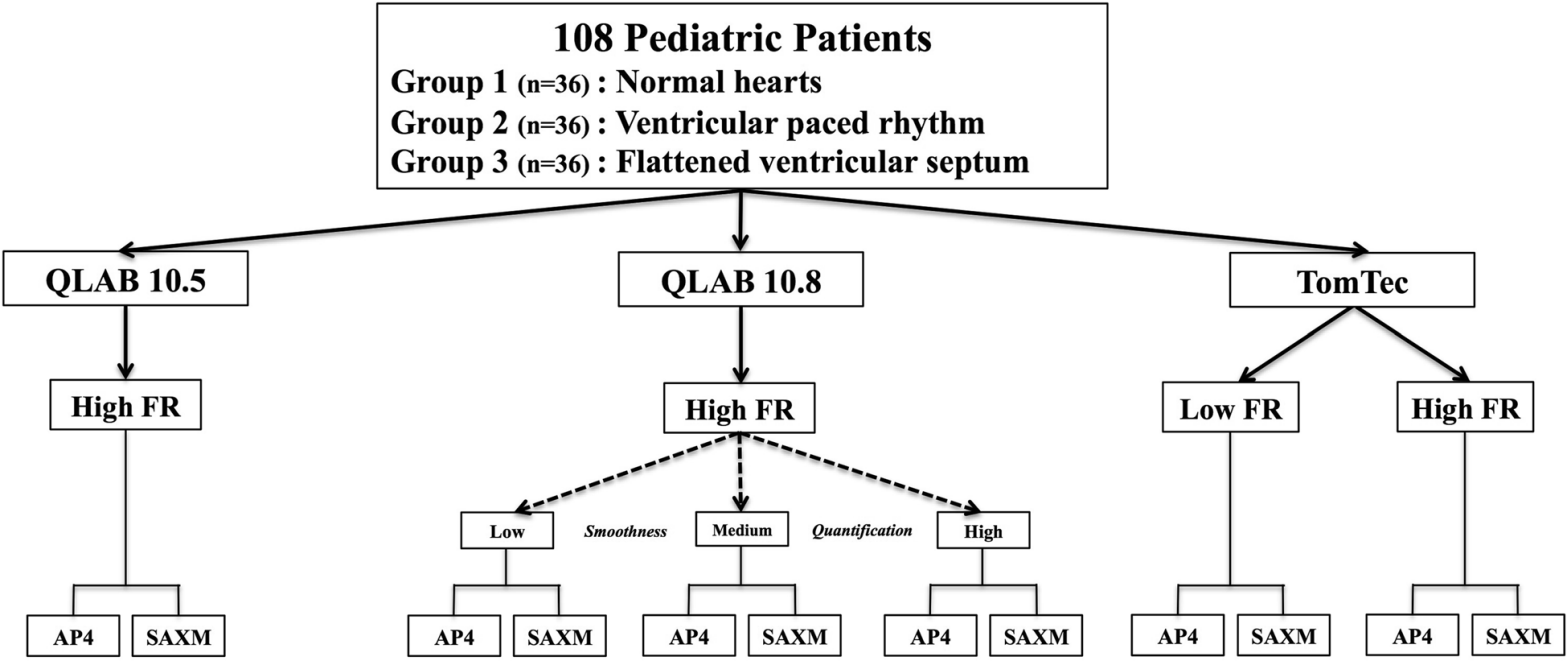
Study methods. One hundred and eight patients were included, with equal representation from 3 groups of children. Analyses were conducted using 2 commercially available vendors and three software packages with a variety of settings (6 variations in total). The same apical 4 chamber (AP4) and parasternal short axis views at the mid-ventricular papillary myscler level (SAXM) views were analyzed for each of the 6 variations. AP4 (Apical four chamber view), FR (frame rate), QLAB 10.8-Low (Low quantification smoothness setting), QLAB 10.8-Med (Medium quantification smoothness settings), QLAB 10.8-High (High quantification smoothness setting), SAXM (Short axis mid-ventricular papillary muscle)

Two-dimensional images were acquired using the Epiq 7 ultrasound system [Philips Medical System, Andover, MA]. Apical 4-chamber (AP4) and short axis views at the mid-papillary level (SAXM) were acquired with optimization performed in each case to best define the endocardial borders. 2D speckle-tracking analysis was performed using two vendors that have been evaluated by the EACVI/ASE task force: QLAB (versions 10.5 and 10.8), and TomTec 2D Cardiac Performance Analysis (2DCPA) version 1.2; of these, only Qlab 10.5 does not fully incorporate the task force recommendations. QLAB 10.5 analysis used an AP4 and SAXM image using the native acquisition frame rate, as it is the only available option. QLAB 10.8 analysis was conducted using high (native) frame rate data from the same selected AP4 and SAXM image with each of the three available settings for quantification smoothness (low, medium, high); these settings modify the degree of spatial averaging of the speckle tracking algorithm. TomTec analysis was performed on the same AP4 and SAXM image using both high (native) frame rate acquisition images and down-sampled Digital Imaging and Communications in Medicine (DICOM) standard (30 frames per second, referred to as ‘low frame rate’). Therefore, in total, 6 variations of software package and settings were investigated using the same source images (Fig. 1).

**Fig. 2:**
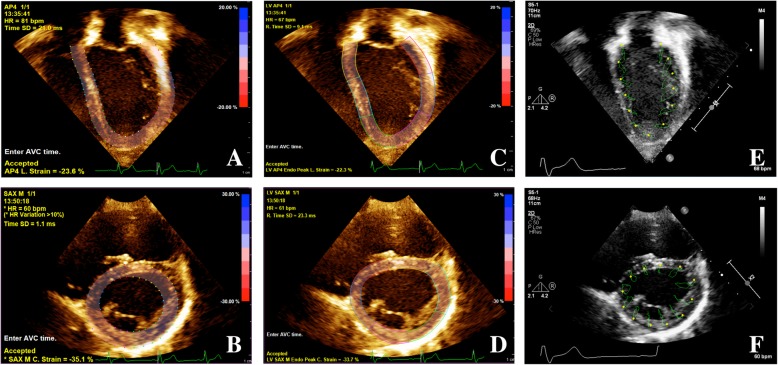
Speckle Tracking Packages Used for Assessment. Screenshots from the various speckle tracking packages. **a**) QLAB 10.5 (AP4 view), **b**) QLAB 10.5 (SAXM view), **c**) QLAB 10.8 (AP4 view), **d**) QLAB 10.8 (SAXM view), **e**) TomTec (AP4 view), **f**) TomTec (SAXM view). AP4 (Apical four chamber view), SAXM (Short axis mid-ventricular papillary muscle)

Speckle tracking analysis was conducted in a standard fashion for each of the software packages. Examples of typical images used for speckle tracking analysis are shown in Fig. 2. Peak negative strain values were recorded. Of note, each of the packages except QLAB 10.5 allow specification and modification of both diastolic and systolic contours (QLAB 10.5 permits modification of the diastolic contour only). Synchrony was calculated as the time to peak (i.e. highest magnitude of strain) standard devation values of the constituent segments [3].

### Intra- and inter-observer reproducibility

Intra- and inter-observer reproducibility were estimated based on data from 15 patients (5 randomly selected from each diagnosis group), in each of the six setting and package combinations. The intra-observer reproducibility was assessed by a single observer (AMF) at least two weeks after the first evaluation to limit recall bias. For inter- and intra-observer reproducibility analysis, the interclass correlation coefficient (ICC) and its 95% confidence interval (CI) was estimated using a one-way analysis of variance model. An ICC < 0.5 was graded as poor, a score > 0.5 to < 0.75 was graded as moderate, a score > 0.75 and < 0.9 was graded as good, and a score ≥ 0.9 was considered to be excellent reliability.

### Tracking quality assessment

Images were classified according to an image grading system defined by our laboratory [11]. The system has five grades of tracking quality: 5) excellent, 4) good, 3) fair, 2) poor, and 1) unusable [17]. For simplicity, we condensed the assessment into three levels: 3) excellent/good (> 2.5), 2) fair (2.0–2.5) and 1) poor/unusable (< 2.0).

### Statistical analysis

A mixed-effects linear regression model with a fixed effect for software package and/or setting and a random effect for subject was used to model mean differences in strain among packages and/or settings. An effect was considered significant if the *p*-value was < 0.05. Formal pairwise comparisons of packages and/or settings were not conducted if the regression model main effect of package and/or setting was not significant at the 0.05 level; *p*-values from pairwise comparisons were not adjusted for multiple comparisons. For strain reproducibility testing, we report the mean absolute difference (because neither value is the standard) and the percentage error, defined as the absolute difference divided by the mean of the two readings, × 100. All quantities were calculated using strain values without the minus sign. All analyses were performed using SAS version 9.4 (SAS Institute, Cary, NC) and R version 3.4.1.

## Results

One-hundred and eight patients were included; subjects’ demographic as well as 2D echocardiographic data are shown in Table 1. Children in Group 2 had lower LV ejection fraction, higher LV end diastolic and systolic volumes, and larger LV mass z scores.

**Table 1:** Demographic and 2D echocardiographic characteristics of participants by age at echocardiogram

	All groups (*n* = 108)	Group 1 (*n* = 36)	Group 2 (*n* = 36)	Group 3 (*n* = 36)	*p*
**Age (y)**	9.5 ± 5.4	9.4 ± 5.4	9.6 ± 5.1	9.5 ± 5.9	0.989
**Female n (%)**	52 (48.2%)	16 (44%)	18 (50%)	18 (50%)	0.913
**Height (cm)**	129.3 ± 33.4	132.2 ± 33.1	128.7 ± 31.1	127.1 ± 36.6	0.805
**Weight (kg)**	33.7 ± 20.9	36.5 ± 22.8	33.4 ± 20.8	31.3 ± 19.1	0.574
**BSA (m**^**2**^**)**	1.08 ± 0.47	1.1 ± 0.5	1.1 ± 0.5	1.0 ± 0.5	0.657
**BMI (kg/m**^**2**^**)**	17.8 ± 3.9	18.5 ± 4.6	17.7 ± 4.1	17.2 ± 3.0	0.339
**SBP (mmHg)**	103.3 ± 12.4	106.7 ± 12.9	102.8 ± 12.1	100.5 ± 11.7	0.101
**DBP (mmHg)**	56.1 ± 8.8	58.1 ± 9	53.4 ± 9.5	56.9 ± 7.4	0.064
**Heart Rate (bpm)**	83.3 ± 24.3	78.4 ± 17.7	83.2 ± 23.6	88.2 ± 29.7	0.238
**2D LVEF (%)**	61 ± 10	65 ± 4	56 ± 13	61 ± 7	**<.001**
**2D LVEF Z score**	−0.6 ± 2.1	0.3 ± 0.9	−1.7 ± 2.9	−0.5 ± 1.5	**<.001**
**2D LVEDV (ml)**	92.8 ± 59.7	87.6 ± 47.0	109.0 ± 71.4	81.7 ± 56.0	0.124
**2D LVEDV Z score**	1.1 ± 2.4	0.4 ± 0.8	2.3 ± 3.1	0.5 ± 2.2	**<.001**
**2D LVESV (ml)**	30.8 (18.3–45.0)	30.6 ± 16.6	53.7 ± 56.5	33.0 ± 29.4	0.021
**2D LVESV Z score**	1.1 ± 2.2	0.2 ± 0.9	2.5 ± 2.6	0.7 ± 2.0	**<.001**
**2D LV mass (gm)**	74.5 ± 47.0	73.5 ± 44.2	84.9 ± 54	65.2 ± 40.8	0.205
**2D LV mass Z score**	0.6 ± 1.8	0.0 ± 0.9	1.6 ± 2.4	0.2 ± 1.4	**<.001**

Values are presented as mean ± SD or median (IQR). Group 1, patients with normal heart structure and function; Group 2, patients with biventricular circulation and ventricular paced rhythm and wide QRS (≥ 120 msec), Group 3, patients with abnormal contour of the ventricular septum in systole or diastole. *p*-value is from Kruskal-Wallis test for 2D LVESV, and from ANOVA (analysis of variance) for all other measures

Abbreviations: *2D* two-dimensional; *BMI* body mass index; *BSA* body surface area; *DBP* diastolic blood pressure; *LVEDV* left ventricular end-diastolic volume; *LVEF* left ventricular ejection fraction; *LVESV* left ventricular end-systolic volume; *SBP* systolic blood pressure; *SD* Standard Deviation; *IQR* Interquartile range

The mean frame rates used for QLAB 10.5, QLAB 10.8 and TomTec high frame rate analyses were 76 ± 15 frames/sec (AP4) and 78 ± 14 frames/sec (SAXM); for the TomTec low frame rate analysis the temporal resolution was 30 frames/ sec.

### Tracking quality assessment and reproducibility

**Fig. 3:**
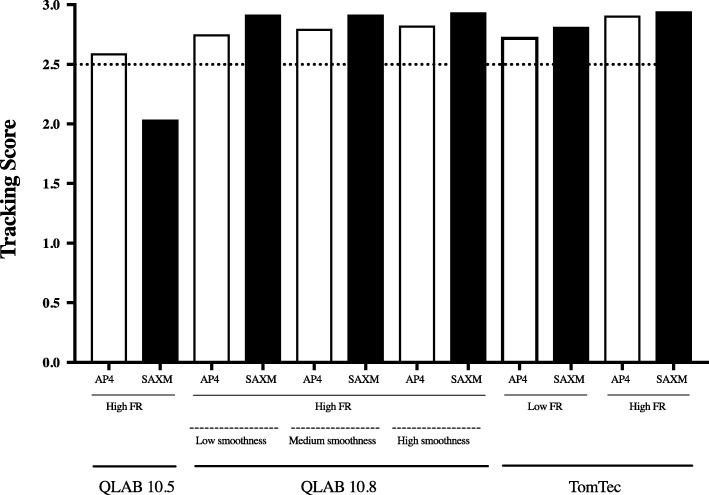
Tracking Assessment Scores. A tracking score was generated for six variations of the software packages included in this study for the AP4 and SAXM views. A tracking score > 2.5 (dotted line) was consider to be excellent. AP4 (Apical four chamber view), FR (frame rate), SAXM (Short axis mid-ventricular papillary muscle)

Tracking score values were high for all packages (all mean value scores > 2.6), except QLAB 10.5, SAXM view (mean score of 2.0) (Fig. 3). Intra- and inter-observer reproducibility results are presented in Table 2 for AP4 and SAXM strain and in Table 3 for AP4 and SAXM TTPSD. ICC values for AP4 and SAXM were good (ICC > 0.75), with the exceptions of the AP4 strain from QLAB 10.8 (medium and high smoothness) and the TomTec low frame rate. For TTPSD, the ICC’s were lower, many in the poor range (< 0.5), with measurements made from AP4 views less reliable than those from SAXM views. The same trends for tracking and reproducibility were observed for each of the individual patient groups and each of three age ranges.

**Table 2:** Strain inter- and intra-observer reproducibility

Measurements (*n* = 15)	Mean difference ± SD	ICC (95% CI)	Mean absolute difference ± SD	% error Median (IQR)
**Inter-observer**				
**QLAB 10.5**				
AP4	0.1 ± 1.5	0.98 (0.95, 0.99)	1.15 ± 0.99	3.46 (0.98–7.79)
SAXM	0.8 ± 3.0	0.92 (0.79, 0.97)	1.99 ± 2.25	5.83 (3.04–10.00)
**QLAB 10.8-Low**				
AP4	0.8 ± 2.2	0.95 (0.85, 0.98)	1.90 ± 1.22	11.38 (6.00–19.61)
SAXM	− 1.8 ± 2.5	0.95 (0.79, 0.98)	2.36 ± 1.91	7.91 (2.95–10.76)
**QLAB 10.8-Med**				
AP4	0.5 ± 1.8	0.96 (0.90, 0.99)	1.61 ± 0.76	9.14 (6.00–20.47)
SAXM	− 1.1 ± 2.6	0.96 (0.88, 0.98)	2.25 ± 1.54	7.10 (4.07–12.64)
**QLAB 10.8-High**				
AP4	0.6 ± 1.8	0.96 (0.90, 0.99)	1.60 ± 0.98	10.74 (4.56–19.76)
SAXM	− 1.5 ± 2.7	0.95 (0.83, 0.98)	2.49 ± 1.74	9.04 (4.78–11.99)
**TomTec-LFR**				
AP4	0.2 ± 4.5	0.80 (0.50, 0.93)	3.55 ± 2.59	17.17 (13.73–24.00)
SAXM	0.3 ± 3.1	0.95 (0.85, 0.98)	2.26 ± 2.03	4.72 (3.40–19.88)
**TomTec-HFR**				
AP4	1.5 ± 2.7	0.91 (0.73, 0.97)	2.58 ± 1.62	15.53 (7.16–25.24)
SAXM	−2.9 ± 2.7	0.92 (0.42, 0.98)	3.17 ± 2.38	11.38 (5.34–17.89)
**Intra-observer**				
**QLAB 10.5**				
AP4	0.6 ± 2.6	0.79 (0.49, 0.92)	1.72 ± 2.02	3.19 (1.31–11.11)
SAXM	0.2 ± 1.8	0.94 (0.84, 0.98)	1.21 ± 1.34	3.10 (1.09–6.52)
**QLAB 10.8-Low**				
AP4	0.7 ± 2.0	0.79 (0.49, 0.92)	1.41 ± 1.59	2.70 (0.98–10.00)
SAXM	− 0.4 ± 2.0	0.94 (0.84, 0.98)	1.75 ± 0.93	5.82 (3.43–8.64)
**QLAB 10.8-Med**				
AP4	0.8 ± 2.5	0.73 (0.37, 0.90)	1.89 ± 1.80	7.55 (3.12–11.45)
SAXM	0.1 ± 2.6	0.90 (0.73, 0.97)	2.07 ± 1.48	6.21 (2.62–10.46)
**QLAB 10.8-High**				
AP4	1.2 ± 2.5	0.73 (0.38, 0.90)	2.00 ± 1.87	5.31 (3.73–14.99)
SAXM	0.1 ± 2.4	0.91 (0.75, 0.97)	2.04 ± 1.06	5.56 (3.81–9.27)
**TomTec-LFR**				
AP4	2.1 ± 2.9	0.65 (0.16, 0.88)	2.73 ± 2.29	11.00 (4.12–19.45)
SAXM	− 1.0 ± 1.6	0.94 (0.79, 0.98)	1.27 ± 1.36	2.47 (0.72–5.32)
**TomTec-HFR**				
AP4	1.0 ± 1.7	0.82 (0.49, 0.94)	1.51 ± 1.26	5.18 (2.84–10.29)
SAXM	0.0 ± 2.0	0.93 (0.79, 0.97)	1.47 ± 1.34	3.58 (0.34–6.45)

Strain values are listed as %. % error is defined as the absolute difference |[Reader 2 − Reader1]|/[median of Reader 1 and Reader 2] × 100

Abbreviations: *AP4* Apical four-chamber view; *CI* Confidence Interval; *ICC* Intraclass Correlation; *IQR* Interquartile Range; *QLAB 10.8 Low* low quantification smoothness setting; *QLAB 10.8 High* high quantification smoothness setting; *QLAB 10.8 Med* medium quantification smoothness setting; *SAXM* short-axis mid papillary muscle level; *SD* Standard Deviation; *TT-30* TomTec at 30 frames per second; *TT-H* TomTec at high frame rate

**Table 3:** Time to Peak Standard Deviation inter- and intra-observer reproducibility

Measurements (*n* = 15)	Mean difference ± SD	ICC (95% CI)	Mean absolute difference ± SD	% error Median (IQR)
**Inter-observer**				
** QLAB 10.5**				
AP4	− 0.5 ± 16.0	0.62 (0.16, 0.85)	11.69 ± 10.44	62.71 (11.34–159.25)
SAXM	2.0 ± 17.9	0.83 (0.56, 0.94)	11.49 ± 13.58	35.72 (23.53–137.50)
**QLAB 10.8-Low**				
AP4	8.4 ± 28.6	0.67 (0.28, 0.87)	21.37 ± 20.13	24.47 (14.89–44.58)
SAXM	5.8 ± 20.0	0.86 (0.64, 0.95)	11.36 ± 17.29	17.72 (2.63–42.43)
**QLAB 10.8-Med**				
AP4	1.7 ± 33.9	0.35 (− 0.21, 0.73)	21.21 ± 25.88	17.09 (7.04–56.85)
SAXM	5.7 ± 27.8	0.74 (0.39, 0.90)	13.59 ± 24.70	10.28 (2.22–77.84)
**QLAB 10.8-High**				
AP4	3.0 ± 24.8	0.53 (0.04, 0.82)	20.26 ± 13.52	79.66 (24.51–98.46)
SAXM	−9.3 ± 19.7	0.81 (0.52, 0.93)	10.90 ± 18.76	37.79 (10.20–58.59)
**TomTec-LFR**				
AP4	−4.3 ± 37.1	0.83 (0.56, 0.94)	24.02 ± 27.95	36.96 (19.78–60.82)
SAXM	6.4 ± 11.9	0.96 (0.87, 0.99)	7.33 ± 11.30	8.37 (1.58–30.17)
**TomTec-HFR**				
AP4	8.5 ± 32.7	0.81 (0.53, 0.93)	18.78 ± 27.72	22.71 (7.60–41.19)
SAXM	9.4 ± 16.8	0.90 (0.69, 0.97)	13.87 ± 13.12	18.85 (4.86–49.68)
**Intra-observer**				
**QLAB 10.5**				
AP4	1.2 ± 10.0	0.79 (0.47, 0.92)	5.75 ± 8.09	20.69 (6.26–83.64)
SAXM	0.2 ± 10.0	0.73 (0.35, 0.90)	4.60 ± 8.41	30.77 (8.13–93.33)
**QLAB 10.8-Low**				
AP4	− 1.3 ± 39.4	0.40 (− 0.15, 0.75)	20.78 ± 33.01	10.30 (5.03–32.49)
SAXM	− 0.8 ± 13.6	0.80 (0.51, 0.93)	7.24 ± 11.33	16.34 (4.88–37.40)
**QLAB 10.8-Med**				
AP4	−1.8 ± 35.8	0.26 (− 0.31, 0.68)	18.40 ± 30.42	13.81 (5.50–48.81)
SAXM	−14.8 ± 54.6	0.18 (− 0.34, 0.62)	18.71 ± 53.28	19.20 (7.00–38.07)
**QLAB 10.8-High**				
AP4	4.5 ± 13.6	0.55 (0.10, 0.81)	7.89 ± 11.87	23.34 (11.04–59.10)
SAXM	− 0.7 ± 15.4	0.50 (− 0.02, 0.80)	6.75 ± 13.70	25.35 (7.13–50.00)
**TomTec-LFR**				
AP4	−13.5 ± 37.7	0.27 (− 0.22, 0.67)	25.59 ± 30.19	38.59 (14.49–106.81)
SAXM	4.6 ± 19.2	0.62 (0.19, 0.85)	11.01 ± 16.17	14.11 (2.22–33.61)
**TomTec-HFR**				
AP4	5.2 ± 19.4	0.57 (0.11, 0.83)	9.87 ± 17.32	16.28 (4.98–21.38)
SAXM	1.6 ± 7.3	0.93 (0.81, 0.98)	6.24 ± 3.71	23.19 (9.00–30.35)

Time to Peak Standard Deviation parameters are expressed as milliseconds. % error represents the absolute difference|[Reader 2 − Reader1]|/[median of Reader 1 and Reader 2] × 100

Abbreviations: *AP4* Apical four-chamber view; *CI* confidence interval; *ICC* inter class correlation; *IQR* interquartile range; *QLAB 10.8 Low* low quantification smoothness setting; *QLAB 10.8 High* High quantification smoothness setting; *QLAB 10.8 Med* medium quantification smoothness setting; *SAXM* short axis mid papillary muscle level; *SD* Standard Deviation; *TT-30* TomTec at 30 frames per second; *TT-H* TomTec at high frame rate

### Strain

**Fig. 4:**
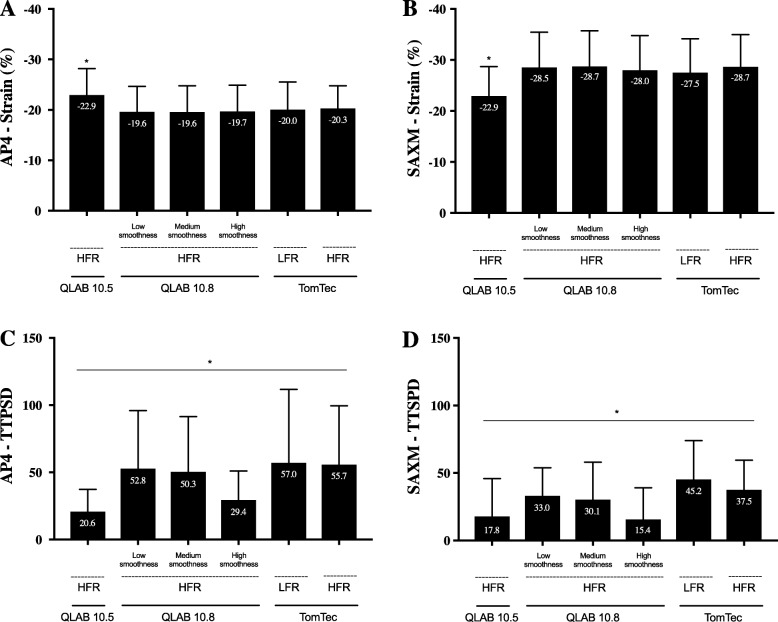
Mean strain and TTPSD values in each software configuration. Bars indicate mean values (±2 standard deviation) in each software configuration based on 108 patients. **a**, AP4 strain; **b**, SAXM strain; **c**, AP4 TTPSD; **d**, SAX-M TTPSD. An asterisk indicates a *p*-value < 0.05. There were many differences in mean AP4 (4C) and SAXM (4D) TTPSD for QLAB 10.5, QLAB 10.8, and TomTec at each frame rate and smoothness setting for the entire cohort. AP4 (Apical four chamber view), HFR (high frame rate), LFR (low frame rate), SAXM (Short Axis Mid papillary muscle); TTPSD (Time To Peak Standard Deviation)

**Fig. 5:**
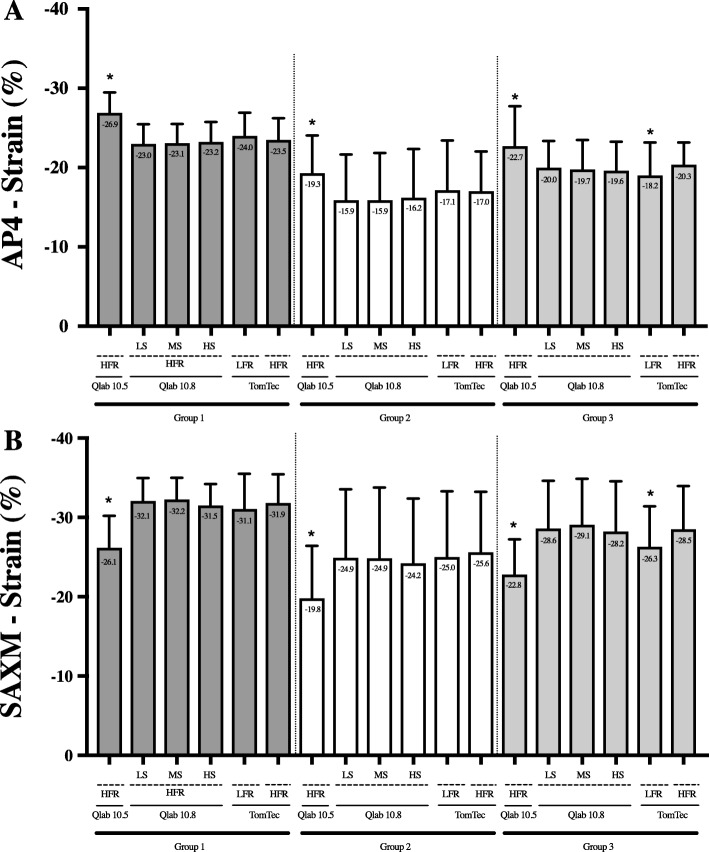
Mean strain values in each software configuration, by group. Mean values are presented (±2 standard deviation) by group (36 patients in each). **a**) AP4 strain; **b**) SAXM strain. An asterisk indicates a *p*-value < 0.05. AP4 Apical four chamber view, FR (frame rate), LS (Low quantification smoothness setting), HS (High quantification smoothness setting), MS (Medium quantification smoothness setting), SAXM (Short Axis Mid papillary muscle). Dark gray bars (group 1) are patients with normal hearts; white bars (group 2) are patients with ventricular paced rhythms; light gray bars (group 3) patients have flattened septal wall

Comparison of AP4 and SAXM strain values among the different vendors (QLAB 10.5 and 10.8, and TomTec) and settings (frame rates and smoothness) for the entire cohort are presented in Fig. 4 with delineation by the three subgroups (all ages combined) in Fig. 5. Considered as an entire cohort (Fig. 4, panels A and B), QLAB 10.5 produced values that were different from the other analysis options, for both AP4 and SAXM measurements. Subgroup analysis (Fig. 5) showed that the mean differences persisted among each of the individual groups. As well, in the flattened ventricular septum group (group 3), the TomTec low frame rate analysis resulted in statistically different mean strain from the other options; this was not true in the other two patient groups.

### TTPSD (synchrony)

**Fig. 6:**
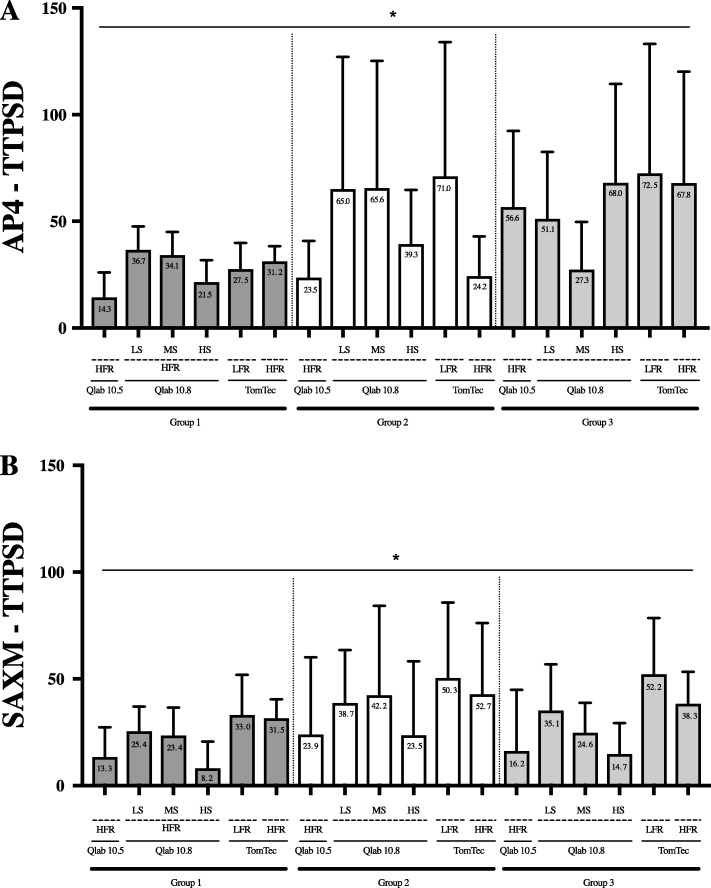
Mean TTPSD values in each software configuration, by group. Mean values are presented (±2 standard deviation) by group (36 patients in each). **a**) AP4 TTPSD; **b**) SAX-M TTPSD. An asterisk indicates a *p*-value < 0.05. There were many differences in mean AP4 and SAXM TTPSD for QLAB 10.5, QLAB 10.8, and TomTec at each frame rate and smoothness setting for all of the subgroups AP4 (Apical four chamber view), LFR (Low frame rate), LS (Low quantification smoothness setting), HS (High quantification smoothness setting), MS (Medium quantification smoothness setting), SAXM (Short Axis Mid papillary muscle). Dark gray bars (group 1) patients have normal hearts; white bars (group 2) patients have ventricular paced rhythms; light gray bars (group 3) patients have flattened ventricular septum.

Comparison of AP4 and SAXM TTPSD and values between the different vendors (QLAB 10.5 and 10.8, and TomTec) and settings (frame rates and smoothness) for the entire cohort are presented in Fig. 4 (panels C and D) with delineation by the three subgroups in Fig. 6. There were many differences in mean AP4 and SAXM TTPSD for QLAB 10.5, QLAB 10.8, and TomTec at each frame rate and smoothness setting, both considered as an entire cohort and divided into subgroups.

## Discussion

In this study we compared longitudinal and circumferential strain and synchrony measures using two commercially available and European Association of Cardiovascular Imaging/American Society of Echocardiography task force tested analysis tools (QLAB and TomTec) with several different settings. The main findings are the following: 1) the tracking feasibility and reproducibility of all measures with QLAB 10.5 differed significantly compared to QLAB 10.8 and TomTec packages (the latter two products fully incorporated the task force recommendations, while the former did not); 2) mean measured time to peak standard deviation (synchrony) differed among all vendors and settings; 3) tracking assessment and strain measures were reproducible and similar among the three smoothness settings for QLAB 10.8; 4) AP4 mean longitudinal and SAXM circumferential strain had minimal differences with good tracking assessment for TomTec at both high and low frame rates in healthy controls and ventricular paced hearts, but showed wider mean differences in patients with flattened interventricular septum at low frame rates. To our knowledge, this is the first study investigating the agreement between commonly used QLAB and TomTec platforms in pediatrics with different smoothness and frame rate settings. The findings in this study imply that the technical characteristics of the tested software packages coupled with the patient population likely have a combined impact on the measurement results.

Few studies have addressed the inter-vendor variability of longitudinal and circumferential strain measurements in children [12, 15, 16, 18, 19]. The EACVI/ASE developed a Task Force to standardize deformation imaging and initially concluded from head-to-head comparison of nine different vendors in adults only (including TomTec Image Arena 2D CPA 1.2 and QLAB version 10.0) that global LV longitudinal strain was feasible and comparable, and in many cases superior to conventional echocardiographic parameters such as volumes and ejection fraction [13]. Prior to the Task Force establishment, Koopman et al. showed that longitudinal strain, measured using a vendor-independent software (TomTec version 1.0) and a vendor-specific software (EchoPAC version 7.0 and QLAB version 7.0) had reasonable agreement, while for circumferential strain wide variability existed between the software packages [15]. Ramlogan et al. recently assessed the measured variability of longitudinal and circumferential strain in children with normal cardiac segmental anatomy and varying cardiac function between two post-EACVI/ASE Task Force recommended software platforms, TomTec (Image-Arena ver. 4.6 SP3 CPA 1.2) and GE (EchoPAC version BT13); and demonstrated reasonable comparison between vendors, with overall longitudinal strain agreement slightly stronger than circumferential [16]. de Waal et al. also reported robust longitudinal strain agreement in term and preterm infants between QLAB 10.8 and TomTec CPA 1.3, but poor correlation with circumferential strain [18]. The data in our study are in line with previous reports in children with respect to similarities in longitudinal strain among vendors, but also suggest a fairly robust agreement with circumferential and longitudinal strain between QLAB 10.8 and TomTec 1.2 in children.

The factors that modulate variability in strain measurement by speckle tracking echocardiography include (1) variability in image acquisition, (2) intra and inter- observer differences in postprocessing, and (3) differences between proprietary software for image analysis; moreover, these differences may be impacted by differences in the pattern of ventricular contraction [11, 20, 21]. Since we analyzed longitudinal and circumferential strain and synchrony from the same AP4 and SAXM image, respectively, for each vendor package and setting, respectively, our design had no bias that may be incurred from use of different images. In order to determine the tracking capability of each software package we utilized a previously validated image-grading system defined by our laboratory [11]. The ability to manually adjust the endocardial border in end-systole in QLAB 10.8 (all three smoothness levels) and TomTec (low and high frame rate), likely resulted in their higher tracking scores, compared to QLAB 10.5. To address postprocessing and software variability, TomTec offers the possibility to perform off-line deformation analysis on archived images acquired by any platform, thus providing a clinically useful tool to analyze and compare studies performed in different centers with different echocardiography platforms. The images imported into TomTec are downsampled to low frame rates, influencing temporal resolution and possible hindering reliable assessment of both peak strain values and timing indices. Low frame rates have also been hypothesized to decrease the agreement of strain and synchrony measurements with other vendors that analyze the images at high frame rates. TomTec also imports images with lower fidelity compared to the raw image files used by the vendor dependent platforms, potentially influencing spatial resolution and decreasing the tracking reliability of the speckle patterns [16]. In our study, we demonstrated that LV longitudinal and circumferential strain showed excellent agreement when using TomTec at high and low frame rates in children with normal cardiac anatomy and ventricular paced rhythm, similar to recent reports in children [15, 16], and adults [17]. For children with abnormal septal contour, however, differences in mean longitudinal and circumferential strain between the high and low frame rate analysis were larger. Given that ventricular volume loading status of the patient does affect the analysis [21], our study demonstrates that it may be rational to obtain both longitudinal and circumferential strain in archived images (low frame rates), but it is inconsistent with the previously suggested notion that archived, low temporal resolution images are adequate for all analyses [21].

Mean strain from all three smoothness settings from QLAB 10.8 were similar. Version 10.7 of the QLAB software was the first to employ the different smoothness settings. A user has the option to preset the quantification (low, medium, or high values) that predefines the assumed smoothness of tissue motion used for the speckle-tracking algorithm by putting the width of a kernel around the tracking points of interest. A higher smoothness quantification setting signifies larger kernels around the region of interest, resulting in less sensitivity of the algorithm to local tissue motion. Our study demonstrated minimal differences in longitudinal and circumferential strain values among the low, medium and high settings for QLAB 10.8, but wide mean differences for TTPSD values. Although we previously demonstrated good reproducibility (intra- and inter- observer reliability) of TTPSD values with QLAB 10.5 at a high frame rate and a single smoothness setting [3], the wider variability of TTPSD using the three different smoothness settings for QLAB 10.8 is not surprising. Whereas the location of the peak of the global strain curve can be adjusted in systole, the boundaries of the constituent segmental curves (determining the TTPSD) cannot be modified accordingly.

Similar to strain, synchrony has been associated with clinical outcomes in children with congenital and acquired heart diseases [7, 8], and a recent study by our echocardiography laboratory identified normal synchrony patterns and z-scores in children [3]. Since ventricular synchrony measures are associated with life-threatening arrhythmias, heart failure, and mortality in pediatric populations [7, 8], understanding the variability between vendors might allow the generalizability of these findings across echocardiography laboratories. Van Everdingen et al. demonstrated minimal differences in longitudinal TTPSD with adults (patients had a range of structurally normal hearts and varying degrees of cardiac function and arrhythmias) between QLAB (10.0) and TomTec (1.2.1.2) [4]. Values were lower (more synchronous) in QLAB compared to the TomTec. In our work, we also observed lower AP4 and SAXM TTPSD means in the QLAB 10.5 and 10.8 (high smoothness setting) compared to TomTec. Although the mechanism for these differences are not completely clear, it is likely that the suboptimal tracking throughout the cardiac cycle (in QLAB 10.5) and spatial averaging effects (in QAB 10.8, high smoothness setting) contribute to an underestimation of dyssynchrony. Overall, we found very wide variation in measures between all the software packages and settings for longitudinal and circumferential TTPSD. Although TTPSD may be reproducible amongst the same vendors in adults [4, 22] and children [3], our data suggest avoidance of interchanging the TTPSD measures between studies using different vendor software packages and settings [3].

### Limitations

The importance of these results should be interpreted within the framework of the inherent limitations in this study. First, while the cardiac cycles analyzed using the different software packages always came from the same acquired clips (typically 2–3 beats in duration), they were not necessarily from the same beat (the beat selected for analysis was the one that was best tracked by the software analysis package); however, given the temporal proximity of all of the beats in the same clip, they would all be expected to reflect a very similar hemodynamic state. Secondly, our study did not analyze the variability with comparison of serial measurements between vendors. Currently, the EACVI/ASE Task Force and other independent studies in infants, children and adults suggest that serial measurements might not be interchangeable when conducting a longitudinal study [15, 18, 19] and “should be preferably interpreted relative to previous examinations with the same machine and software” [13]. In practice “Global” LV strain has been defined by different LV segmentation models that assesses either an 18-, 17- or 16- segment model from the averaging of the three apical views (for longitudinal strain) and the three short axis views (for circumferential strain) [10, 13, 23]. In children deformation imaging is often reported only from the AP4 view for longitudinal strain and SAXM for circumferential [1, 2, 13] because it is sometimes difficult to show an apex clearly as it exists near the surface and it is not always contained in the view [24]. Moreover, not every package that can be used in pediatrics was included in this study; however, the products chosen represent some of the most popular and commonly used tools available. Finally, no attempt was made to directly compare the values or reproducibility of regional strain measurements; however the analysis of ventricular synchrony that was performed in this work does address issues of regional functional assessment.

## Conclusions

This study investigated the agreement between commonly used QLAB and TomTec platforms in pediatrics with different smoothness and temporal resolution settings. Mean synchrony (TTPSD) varied substantially among all QLAB and TomTec packages and settings in children. Mean longitudinal (AP4) and circumferential (SAMX) strain also differed between QLAB 10.5 and all other software packages, irrespective of the temporal or spatial resolutions, suggesting avoidance of QLAB 10.5 comparisons with contemporary QLAB versions and vendor-independent TomTec software. For QLAB 10.8, the various quantification smoothness settings resulted in minimal variability in SAXM and AP4 views. Compared with QLAB 10.8, there were also minimal differences with TomTec for both longitudinal and circumferential strain values, even with the high and low frame rate settings, for the normal and ventricular paced cohorts; for children with abnormal septal contours, mean differences according to package/setting were greater. These findings suggest that reliable comparisons among these tools, using different settings, may be appropriate in many settings, but clinicians and researchers should use these tools with caution.

## Data Availability

The data and material are available upon request with Institutional approval from our IRB.

## Abbreviations

AP4: Apical four chamber view
BMI: Body mass index
BSA: Body surface area
CI: Confidence interval
DBP: Diastolic blood pressure
FR: Frame rate
HFR: High frame rate
ICC: Interclass correlation coefficient
IQR: Interquartile range
LFR: Low frame rate
LV: Left ventricle
LVEDV: Left ventricular end diastolic volume
LVEF: Left ventricular ejection fraction
LVESV: Left ventricular end systolic volume
SAXM: Short axis at mid-papillary level
SBP: Systolic blood pressure
SD: Standard deviation
TTPSD: Time to peak standard deviation
2D: Two-dimensional
